# Anti-Inflammatory Effects of Melandrii Herba Ethanol Extract via Inhibition of NF-κB and MAPK Signaling Pathways and Induction of HO-1 in RAW 264.7 Cells and Mouse Primary Macrophages

**DOI:** 10.3390/molecules21060818

**Published:** 2016-06-22

**Authors:** Yun Hee Jeong, You-Chang Oh, Won-Kyung Cho, Bohyoung Lee, Jin Yeul Ma

**Affiliations:** Korean Medicine (KM)-Application Center, Korea Institute of Oriental Medicine, 70, Cheomdanro, Dong-gu, Daegu 41062, Korea; runxi0333@kiom.re.kr (Y.H.J.); ulivuli@kiom.re.kr (Y.-C.O.); wkcho@kiom.re.kr (W.-K.C.); leebh@kiom.re.kr (B.L.)

**Keywords:** Melandrii Herba, inflammatory response, heme oxygenase-1, nuclear factor-κB, mitogen-activated protein kinase

## Abstract

Melandrii Herba (MH) is a traditional Asian medicinal herb used to treat breast cancer, anuria, and diseases of lactation. However, its biological properties and molecular mechanisms have not been fully elucidated. The purpose of this study was to investigate the anti-inflammatory activity and underlying molecular mechanism of MH ethanol extract (MHE) on the lipopolysaccharide (LPS)-mediated inflammatory response in macrophages. MHE cytotoxicity was determined using a cell counting kit (CCK) assay. The effects of MHE on the production of NO, inflammatory cytokines, and related proteins and mRNAs were determined using the Griess test, ELISA, Western blotting, and real-time RT-PCR, respectively. In addition, intracellular signaling pathways, such as NF-κB, MAPK, and HO-1, were analyzed using Western blotting. Our results revealed that MHE treatment significantly inhibited the secretion of NO and inflammatory cytokines, including TNF-α, IL-6, and IL-1β in macrophages, at sub-cytotoxic concentrations. Furthermore, MHE treatment inhibited iNOS expression and induced HO-1 expression. Finally, the transcriptional activities of NF-κB and MAPK activation were significantly suppressed by MHE in LPS-stimulated macrophages. The results indicate that MHE exerts anti-inflammatory effects by suppressing inflammatory mediator production via NF-κB and MAPK signaling pathways inhibition and induction of HO-1 expression in macrophages. Therefore, our results suggest the potential value of MHE as an inflammatory therapeutic agent developed from a natural substance.

## 1. Introduction

Melandrii Herba (MH), or “wangbulruehaeng” in Korean, is the aboveground portion of fruiting *Melandryum firmum* Rohrbach (Caryophyllaceae). *M. firmum* grows in Asia and is primarily produced in Korea, China, and Japan. MH is an important traditional Asian medicinal herb used to treat breast cancer, anuria, and diseases of lactation [1]. In the Shennong′s Classic of Materia Medica, MH is reported to promote lactation and have diuretic and hemostatic effects. The main compounds in MH include sapogenins, saponins, triterpenoids, and flavonoids [2,3,4], which have been evaluated for bioactivity. However, the effects of MH on inflammation and its molecular mechanism of action remain to be elucidated.

Inflammation is important in host response to harmful stimuli, such as tissue damage, viral infection, chemical exposure, and trauma. During inflammatory processes, macrophages are crucial in regulating the innate immune response and primary target cells of lipopolysaccharide (LPS). LPS, which are endotoxins, comprise the major component of the outer membrane of Gram-negative bacteria and activate macrophages to release various inflammatory mediators, such as nitric oxide (NO), inducible nitric oxide synthase (iNOS), cyclooxygenase (COX)-2, and inflammatory cytokines [5,6]. Excess production of these pro-inflammatory mediators causes various inflammatory diseases, including rheumatoid arthritis, asthma, atherosclerosis, vascular diseases, inflammatory bowel disease, and cancer [7,8,9,10,11,12].

Inflammatory mediators are regulated by several transcription factors and signaling pathways. For example, nuclear factor (NF)-κB is composed mainly of p50 and p65 subunits in mammalian cells. In unstimulated states, p65 is present in an inactive form in the cytoplasm bound to its suppressor protein, inhibitor of NF-κB (IκB). After activation by inflammatory stimulants, the IκB–kinase complex is rapidly phosphorylated and degraded. The resulting free NF-κB migrates into the nucleus, where it activates transcription of target genes and promotes pro-inflammatory mediators, including iNOS, COX-2, and certain cytokines [13,14,15,16]. NF-κB pathway activation is associated with phosphorylation of mitogen-activated protein kinase (MAPK), such as extracellular regulated kinase (ERK), c-Jun N-terminal kinase (JNK), and the p38 subfamily. The MAPK family also regulates the inflammatory molecules and immune-related cytotoxic factors that induce inflammatory gene expression in macrophages [17,18,19,20]. Furthermore, many studies have demonstrated that MAPKs are important in activating the NF-κB signaling pathway [21,22,23]. Therefore, NF-κB and MAPKs are key targets for treating various inflammatory diseases.

NO, a gaseous free radical produced by iNOS, has been identified as a regulatory mediator involved in the process of various inflammatory diseases [24]. In addition, iNOS expression is associated with heme oxygenase (HO)-1 induction. HO is the rate-limiting enzyme for heme degradation and has three isoforms, HO-1, HO-2, and HO-3. Among them, only HO-1 decreases pro-inflammatory mediator expression, such as NO, tumor necrosis factor (TNF)-α, interleukin (IL)-6, and IL-1β, in stimulated macrophages. Therefore, many therapeutic agents present anti-inflammatory effects via HO-1 overexpression.

In this study, we evaluated the inhibitory effect of MH ethanol extract (MHE) on inflammatory response by LPS stimulation in mouse macrophages. In addition, we determined whether the effects of MHE on NF-κB and MAPK signaling pathways, as well as the induction of HO-1 expression, explain the anti-inflammatory mechanism of MHE. We also investigated chemical constituents of MHE via high-performance liquid chromatography (HPLC) analysis.

## 2. Results

### 2.1. Effects of MHE on Cell Viability

**Figure 1:**
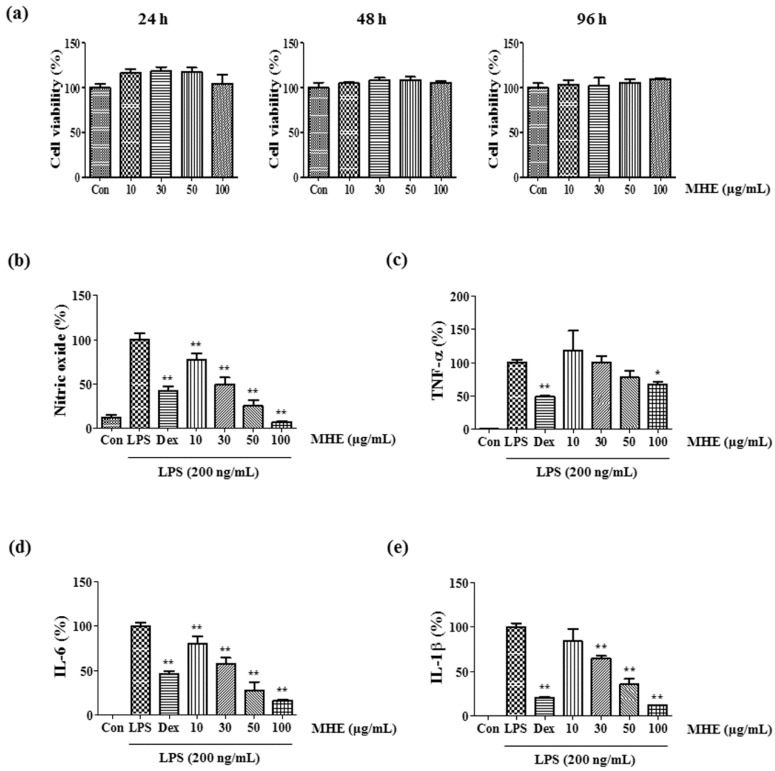
Effects of MHE on (**a**) cell viability and (**b**–**e**) the production of inflammatory mediators in RAW 264.7 cells. Cells were pretreated with MHE for 1 h, followed by a 24 h incubation with LPS. (**a**) Cell viability was measured using a CCK assay; (**b**) NO content in conditioned medium was determined using Griess reagent; (**c**–**e**) Cytokine levels in the medium were measured using ELISA. Con: control; Dex: 10 μM dexamethasone. As a control, cells were incubated with the vehicle alone. * *p* < 0.01 and ** *p* < 0.001 were calculated from comparing with LPS-stimulation value.

The cytotoxicity of MHE in RAW 264.7 cells was evaluated using the cell counting kit (CCK) assay after the 24–96 h treatment to determine the optimal concentration that would be effective for anti-inflammation with minimum toxicity. The results showed that concentrations of MHE ≤ 100 μg/mL had no significant effects on cell viability, indicating MHE is not toxic to cells (Figure 1a). Therefore, subsequent experiments were performed with concentrations ≤100 μg/mL.

### 2.2. Inhibitory Effects of MHE on NO Production

Since NO production is correlated with various inflammatory diseases, we determined the suppressive effects of MHE on NO levels in macrophages via LPS stimulation. To determine NO levels in the supernatant, cells were pretreated with 10–100 μg/mL of MHE for 1 h, followed by stimulation with LPS for 24 h, and then measured using Griess reagent. As the positive control, dexamethasone (10 μM) shows strong suppressive effect on NO secretion upon LPS stimulation. Additionally, MHE dramatically inhibited NO production in a dose-dependent manner after LPS stimulation (Figure 1b). Especially, MHE shows stronger inhibitory effect more than positive control (10 μM dexamethasone) on NO secretion at concentrations of 50 and 100 μg/mL.

### 2.3. Effects of MHE on Inflammatory Cytokines and mRNA Expression

**Figure 2:**
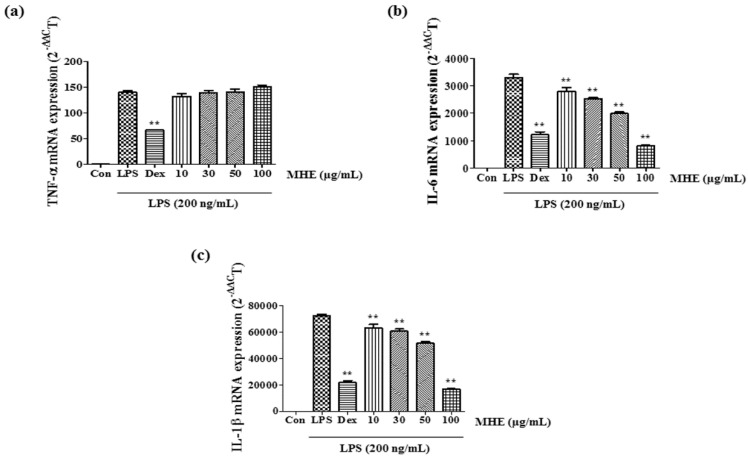
Effects of MHE on LPS-induced (**a**) TNF-α; (**b**) IL-6; and (**c**) IL-1β mRNA expression in RAW 264.7 cells. Cells were pretreated with MHE for 1 h and stimulated with LPS for 6 h. mRNA levels were measured using real-time RT-PCR. ** *p* < 0.001 was calculated from comparing with LPS-stimulation value.

The effects of MHE on inflammatory cytokine secretion including TNF-α, IL-6, and IL-1β and their mRNA expression in macrophages were evaluated using enzyme-linked immunosorbent assay (ELISA) and real-time reverse transcription-polymerase chain reaction (RT-PCR), respectively. IL-6 and IL-1β secretion were significantly inhibited by MHE in a dose-dependent manner (Figure 1d,e). Also, inhibitory rates were reached 83% and 88% (IL-6 and IL-1β cytokines, respectively) at a concentration of 100 μg/mL. However, TNF-α production was inhibited only slightly at the highest MHE concentration (100 μg/mL) (Figure 1c). Consistent with these results, MHE treatment significantly inhibited IL-6 and IL-1β mRNA expression in a dose-dependent manner (Figure 2b,c). However, MHE had no effect on TNF-α mRNA expression at all concentrations (Figure 2a).

### 2.4. Effects of MHE on iNOS, COX-2, and HO-1 Expression

**Figure 3:**
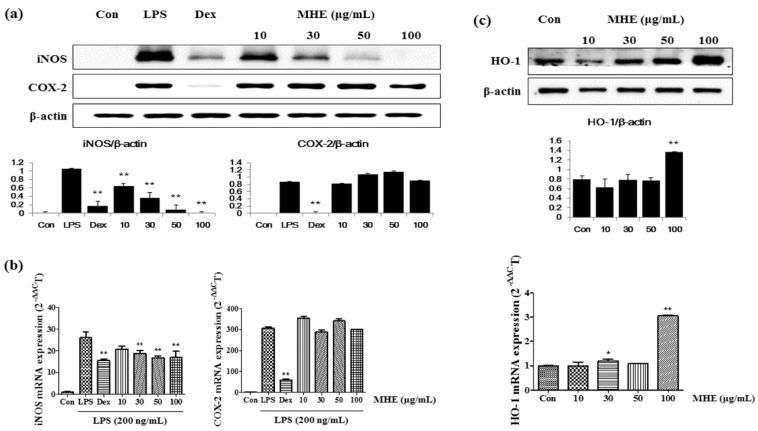
Effects of MHE on (**a**) iNOS and COX-2 protein levels; (**b**) mRNA expression; and (**c**) HO-1 in RAW 264.7 cells. Cells were treated with (**a**,**b**) LPS alone or LPS and MHE for 24 h, respectively, and (**c**) MHE alone for 6 h. β-actin served as the control. (**a**,**c**) Each histogram shows protein expression value compared with β-actin of same sample. The experiment was repeated three times independently, and similar results were obtained. * *p* < 0.01 and ** *p* < 0.001 were calculated from comparing with LPS-stimulation value.

The effects of MHE on iNOS, COX-2, and HO-1 protein levels and mRNA expression in RAW 264.7 cells were investigated using Western blots and real-time RT-PCR analysis. MHE treatment significantly decreased iNOS protein levels in a dose-dependent manner (Figure 3a). In particular, treatment of 50 and 100 μg/mL MHE inhibited iNOS expression nearly 91%–98%. However, COX-2 protein levels were not influenced by MHE treatment at all concentrations. Consistent with the results of protein, MHE inhibited expression of iNOS mRNA but not COX-2 mRNA levels (Figure 3b). However, inhibitory activity of MHE on iNOS mRNA expression was lower compared with protein result. Additionally, HO-1 protein levels and mRNA expression increased after MHE treatment at a concentration of 100 μg/mL (Figure 3c). In the previous study, natural herbal extract induced HO-1 expression in macrophages and it influenced regulation of other inflammatory mediators [25,26]. Therefore, in the present study, we demonstrated the effect of MHE on HO-1 induction and its influence on inhibition of inflammatory mediator production, such as NO and iNOS.

### 2.5. Effects of MHE on LPS-Induced NF-κB Nuclear Translocation and IκBα Phosphorylation in Macrophages

**Figure 4:**
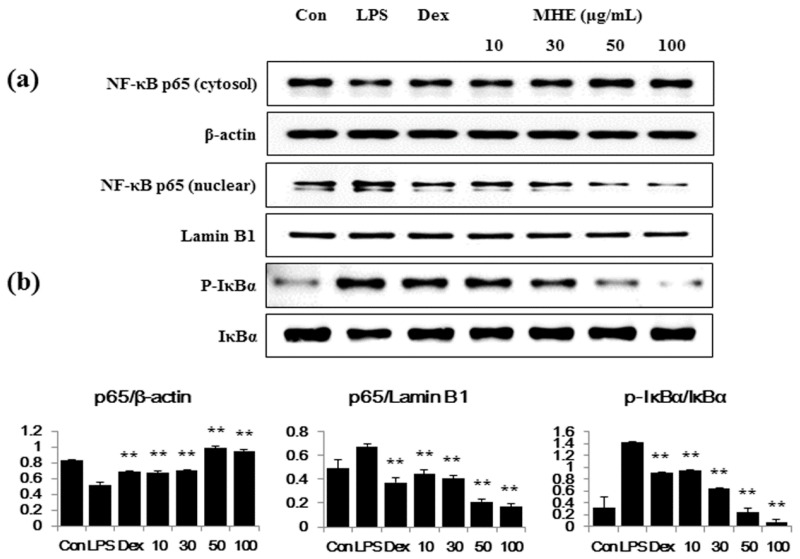
Effects of MHE on (**a**) NF-κB p65 translocation into the nucleus and (**b**) IκBα phosphorylation in RAW 264.7 cells. Cells were pretreated with MHE for 1 h and stimulated with LPS for an additional (**a**) 1 h or (**b**) 30 min. β-actin and Lamin B1 were used to measure the cytosolic and nuclear control proteins, respectively. Each histogram shows the protein expression value compared with the control protein of the same sample. The experiment was repeated three times independently and similar results were obtained. ** *p* < 0.001 was calculated from comparing with LPS-stimulation value.

We examined the effects of MHE on LPS-induced changes in the transcription factors NF-κB, phospho-IκBα, and IκBα, as they are important in regulating the inflammatory process. As shown in Figure 4a, MHE greatly obstructed the LPS-induced reduction of cytosol p65 and nuclear translocation of p65 (reduce nuclear p65) in a dose-dependent manner. β-actin and Lamin B1 were used as a control protein, respectively. Consistent with the results of p65, MHE suppressed IκBα degradation and phosphorylation in the cytoplasm in a dose-dependent manner (Figure 4b). These results suggested that MHE treatment effectively blocked LPS-induced NF-κB activation via inhibited degradation of IκBα in macrophages. Correlation between the blockade of some herbal extract on NF-κB pathway activation and its inhibitory effect on the production of inflammatory molecules, including cytokines, was demonstrated in a previous study [16]. Thus, we demonstrated a suppressive effect of MHE on NF-κB pathway activation and its direct effect on the regulation of inflammatory response.

### 2.6. Effects of MHE on MAPK Phosphorylation in LPS-Stimulated Macrophages

**Figure 5:**
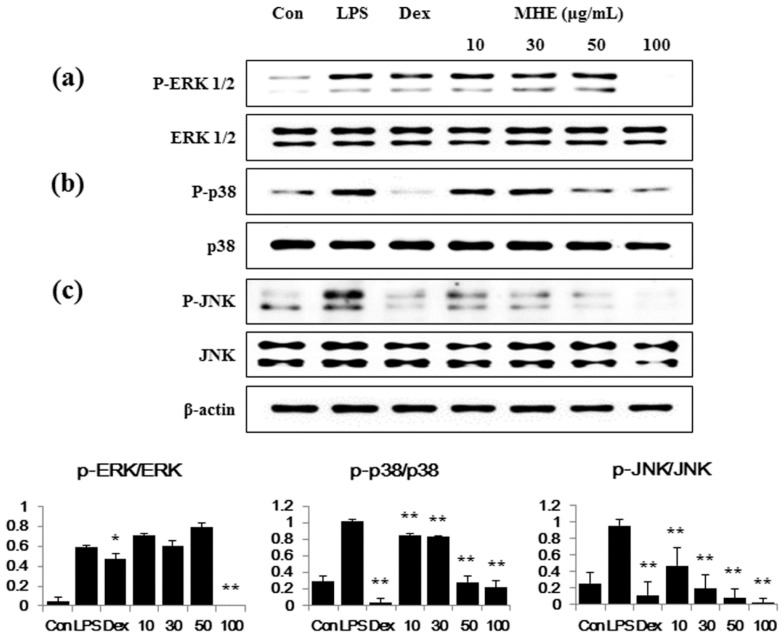
Effects of MHE on the phosphorylation of (**a**) ERK; (**b**) p38; and (**c**) JNK MAPKs in RAW 264.7 cells. Cells were treated with MHE for 1 h and exposed to LPS for 30 min. ERK, p38, JNK, and their phosphorylated forms (P-ERK, P-p38, and P-JNK) were examined using Western blot analyses. Each histogram shows the phosphorylation value compared with the non-phospho protein of the same sample. The experiment was repeated three times independently, and similar results were obtained. * *p* < 0.01 and ** *p* < 0.001 were calculated from comparing with LPS-stimulation value.

Since the MAPKs ERK1/2, p38, and JNK are essential in mediating inflammatory response [27], we examined the effects of MHE on MAPK activation with Western blot analyses using specific antibodies to detect their phosphorylated forms. As shown in Figure 5b,c, MHE treatment strongly inhibited p38 and JNK phosphorylation at all concentrations. Additionally, ERK phosphorylation was inhibited only at the highest MHE concentration (100 μg/mL) (Figure 5a). However, phosphorylation of ERK perfectly suppressed by 100 μg/mL MHE treatment.

### 2.7. Effects of MHE on Inflammatory Cytokine Production in LPS-Induced Mouse Peritoneal Macrophages

**Figure 6:**
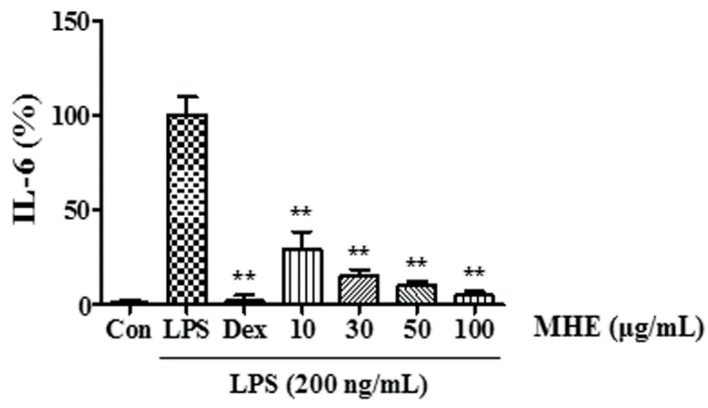
Effect of MHE on IL-6 production in LPS-stimulated mouse peritoneal macrophages. Cells were treated with MHE for 1 h and stimulated with LPS for 24 h. Cytokine production was measured using ELISA. ** *p* < 0.001 was calculated from comparing with LPS-stimulation value.

We examined the anti-inflammatory effect and its inhibitory mechanism of MHE on LPS-stimulated RAW 264.7 cells and confirmed its inhibitory effect in mouse primary macrophages. We measured the levels of three inflammatory cytokines, TNF-α, IL-6, and IL-1β. As shown in Figure 6, MHE significantly inhibited IL-6 secretion in a dose-dependent manner. However, MHE did not inhibit TNF-α or IL-1β secretion. These results indicated that MHE effectively inhibited the inflammatory response in primary macrophages without toxicity.

### 2.8. Representative Chromatograms of the Constituents in MHE

**Figure 7:**
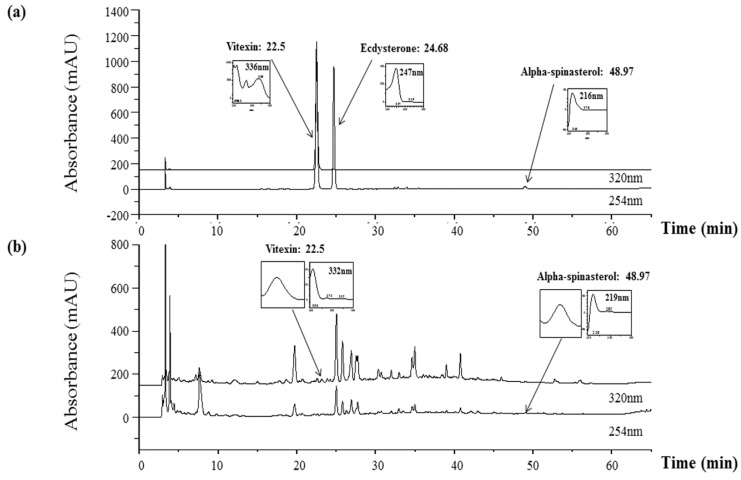
HPLC-DAD analysis of three constituents in MH. Vitexin, ecdysterone, and α-spinasterol were identified in (**a**) the standard mixture and (**b**) MHE at wavelengths of 250 nm and 320 nm.

The constituents of MH were determined with HPLC analysis, and each peak of the UV spectra was compared with that of the representative standard compounds. The HPLC-DAD analysis revealed two compounds in the MHE based on their UV spectra and retention times, vitexin (332 nm, tR: 22.5 min), and α-spinasterol (217 nm, tR: 48.99 min) (Figure 7). Conversely, ecdysterone was not detected in MHE.

## 3. Discussion

MH is an important traditional medicinal herb belonging to Caryophyllaceae, and is commonly used to treat breast cancer, anuria, and diseases of lactation [1]. In this study, we investigated the anti-inflammatory effects and underlying mechanism of MHE in LPS-stimulated RAW 264.7 cells and mouse primary macrophages. We first examined influence of MHE treatment on macrophage viability at 24, 48 and 96 h using CCK assay. MHE (up to 100 μg/mL) did not affect the viability of macrophages at 24, 48, or 96 h treatment, respectively. Therefore, the following experiments were performed with four concentrations ≤100 μg/mL. Cells were pretreated with various concentrations (10–100 μg/mL) of MHE and then stimulated with LPS (200 ng/mL). In inflammatory processes upon LPS stimulation, the pro-inflammatory mediators NO and prostaglandin (PG)E_2_ are generated by iNOS and COX-2, respectively [28]. Therefore, we examined the influence of MHE treatment on the production of NO, iNOS, and COX-2 induced by LPS exposure. MHE significantly inhibited NO secretion and strongly suppressed iNOS protein levels and mRNA expression in a dose-dependent manner, but it did not affect COX-2 expression. In addition, we investigated the effect of MHE treatment without LPS on HO-1 protein levels and mRNA expression, as induction of HO-1 signal transduction is closely associated with NO and iNOS production. As a result, MHE significantly induced HO-1 expression in a concentration of 100 μg/mL, both protein and mRNA level. These results indicated MHE treatment induced HO-1 expression in macrophages and it affected the inhibiting efficacy of NO and iNOS production.

Several pro-inflammatory cytokines, including TNF-α, IL-6, and IL-1β, are critical in the innate immune response. Therefore, we examined the effect of MHE on LPS-induced TNF-α, IL-6, and IL-1β production and found that MHE resulted in a dose-dependent reduction on IL-6 and IL-1β in LPS-stimulated RAW 264.7 cells. However, MHE only slightly inhibited TNF-α production at the highest concentration. Additionally, MHE strongly inhibited IL-6 production in mouse primary macrophages after LPS stimulation. Pro-inflammatory cytokine transcription is regulated by transcriptional factors, such as NF-κB [29,30,31]. The NF-κB transcription factors are known to be a critical molecule for immunity and LPS-stimulated inflammation [32] and are regarded as main therapeutic agents in the treatment of various inflammatory diseases. Members of the NF-κB family, including NF-κB1 (p105/p50), NF-κB2 (p100/p52), p65 (Rel A), c-Rel, and RelB [33,34]. Among them, the p65/p50 heterodimer is the predominant combination involved in transcriptional activation processes. In unstimulated state, p65 subunits of NF-κB is sequestered by inhibitors of NF-κB alpha (IκBα) in the cytoplasm [35,36]. Activation of NF-κB by the stimulation of LPS, exists via ultimate degradation of IκB bound to NF-κB, leading to increases the translocation of NF-κB into the nucleus, where they promote transcription of pro-inflammatory factors [37,38,39]. In addition, activation of other such pathways, including MAPK and PI3K/AKT, is also involved in the stimulation of NF-κB [35,36]. In this study, we measured p65 levels in cytoplasmic and nuclear extracts and investigated phosphorylated IκBα levels using Western blotting. MHE reduced NF-κB p65 subunit nuclear translocation by blocking LPS-induced IκBα phosphorylation and degradation in a dose-dependent manner. This result indicates that NF-κB transcription to the nucleus is decreased by MHE treatment.

The MAPKs are serine-threonine kinases that mediate a significant number of cellular activities, such as gene expression, mitosis, proliferation, cell survival, and extracellular signal transduction to the nucleus [40,41,42]. There are three major subgroups of MAPK, including ERK, JNK, and p38 are also important signaling pathway involved in controlling immune responses and inflammatory factors in RAW 264.7 macrophages [36,43]. The phosphorylation of MAPKs by LPS-stimulation are known to be induces NF-κB activation and initiates pro-inflammatory responses [44,45,46,47]. Therefore, we examined whether MHE treatment suppressed MAPK phosphorylation and found that MHE efficiently inhibited the phosphorylation of p38 and JNK in a dose-dependent manner. Additionally, MHE perfectly inhibited ERK phosphorylation at the highest concentration. These results demonstrate that MAPK signaling pathway activation is inhibited by MHE and that inhibition of MAPK activation is closely associated with the suppression of pro-inflammatory mediator production and the reduction in NF-κB activation in RAW 264.7 macrophages.

We confirmed two main components in MHE, vitexin and α-spinasterol (Figure 7), which is consistent with the results presented in previous reports [48]. Vitexin was reported to exert a cardioprotective effect by regulating inflammatory cytokines and the MAPK pathway in ischemia/reperfusion injury in rats [49]. In another study, vitexin inhibited inflammatory pain by targeting TRPV1, oxidative stress, and cytokines in mice [50]. A recent study found that α-spinasterol had anti-inflammatory activity in LPS-induced peritonitis in mice [51]. This suggests that the anti-inflammatory activity of MHE might be related to bioactive compounds in MHE, including vitexin and α-spinasterol. However, additional studies on other compounds and on the biological activities of MH are necessary.

## 4. Experimental Section

### 4.1. Plant Material

Dried whole MH was provided by Yeongcheon Hyundai Herbal Market (Yeongcheon, Korea) and identified by Prof. KiHwan Bae, Chungnam National University, Korea. To prepare the MHE, dried MH powder (30.0 g) was extracted with 390 mL of 70% ethanol at 40 °C in a shaking incubator for 24 h. After extraction, the solution was filtered using 185 mm filter paper (Whatman, Piscataway, NJ, USA). The ethanol filtrate was concentrated using a rotary vacuum evaporator (Buchi, Tokyo, Japan), and 3.0286 g were acquired, with a yield of 10.0953%. Samples were freeze-dried and maintained in desiccators at −20°C before use.

### 4.2. Reagents and Cell Culture

The murine macrophage cell line RAW 264.7 was obtained from American Type Culture Collection (ATCC, Manassas, VA, USA). RPMI 1640 medium, fetal bovine serum (FBS), and antibiotics were purchased from Lonza (Basel, Switzerland). LPS, bovine serum albumin (BSA), and dexamethasone were obtained from Sigma-Aldrich (St. Louis, MO, USA). Cell counting kits (CCK) and ELISA antibody assays were obtained from Dojindo Molecular Technologies (Kumamoto, Japan) and eBioscience (San Diego, CA, USA), respectively. The primary and horseradish peroxidase-conjugated secondary antibodies used in this study were purchased from Cell Signaling Technology, Inc. (Boston, MA, USA) and Santa Cruz Biotechnology (Santa Cruz, CA, USA). Nitrocellulose (NC) membranes were acquired from Millipore (Bedford, MA, USA). RNA extraction kits, DNA synthesis kits, oligonucleotide primers, and AccuPower^®^ 2X Greenstar qPCR Master Mix (ROX) were purchased from iNtRON Biotechnology (Daejeon, Korea) and Bioneer (Daejeon, Korea). Murine macrophage RAW 264.7 cells were cultured in complete RPMI 1640 medium supplemented with 100 U/mL of penicillin, 100 μg/mL of streptomycin, and 10% heat-inactivated FBS at 37 °C in a humidified incubator with 5% CO_2_.

### 4.3. Preparation of Mouse Peritoneal Macrophages

Male BALB/c mice (25 ± 3 g) were purchased from Samtako Bio Korea (Osan, Korea). To obtain mouse peritoneal macrophages, mice were treated with 300 μL of sterile 3% sodium thioglycolate (Sigma-Aldrich) three days before sacrificing via cervical dislocation. Animals were housed five per cage in an air-conditioned room at 22 °C–24 °C with a 12 h:12 h light/dark cycle and were allowed food and water ad libitum. Peritoneal macrophages were harvested by washing the peritoneal cavity with 10 mL of ice-cold phosphate buffered saline (PBS) and massaging the abdomen gently for 3 min. The peritoneal fluid was drawn out and centrifuged at 1000 rpm for 5 min at 4 °C, and the supernatant was discarded. The cells were re-suspended in complete RPMI 1640 medium and incubated for 18 h in cell culture plates. Then, the medium was exchanged with fresh serum-free RPMI 1640, and LPS was added in the presence or absence of MHE for 24 h. All animal studies were performed following the Guide for the Animal Care and Use Committee of the Korea Institute of Oriental Medicine (permission number # 14-079).

### 4.4. Cell Viability Using the CCK Assay

The cytotoxic effects of MHE were assessed in the absence or presence of LPS using the CCK assay. Briefly, RAW 264.7 cells were plated at a density of 5 × 10^5^ cells/mL in 96-well culture plates and incubated at 37 °C for 18 h. The cells were treated with various concentrations of MHE. After the 24 h incubation, the CCK solution was added to each well, and the cells were incubated for 1 h. Wells were measured for absorbance at 450 nm using an ELISA reader (Infinite M200, Tecan, Männedorf, Switzerland).

### 4.5. Nitrite Concentration Using the Griess Test

The nitrite concentration in the supernatant from cultured macrophage cells was analyzed using the Griess reaction test. RAW 264.7 cells were plated at a density of 5 × 10^5^ cells/mL in 96-well culture plates, incubated with MHE for 1 h, and stimulated with LPS for 24 h. Griess reagent (1% sulfanilamide, 0.1% *N*-1-napthylethylenediamine dihydrochloride, and 2.5% phosphoric acid) was mixed with an equal volume of cell supernatant, and absorbance was measured at 570 nm using the ELISA reader. Sodium nitrite was used as a standard. Dexamethasone, which is widely used for the treatment of inflammation-related disease, was used as a positive control in this study (10 μM).

### 4.6. Cytokine Production Using ELISA Assays

The concentrations of the inflammatory cytokines TNF-α, IL-6, and IL-1β in culture supernatant were determined using ELISA antibody kits following the manufacturer′s protocol. RAW 264.7 cells were grown in 24-well culture plates at a density of 5 × 10^5^ cells/mL, incubated with MHE for 1 h, and stimulated with LPS for 24 h. The cytokines produced in each sample were calculated from standard curves using known concentrations of recombinant cytokines for each ELISA antibody kit.

### 4.7. Preparation of Whole Cell, Cytosolic, and Nuclear Fractions

RAW 264.7 cells were seeded at 1 × 10^6^ cells/mL in six-well culture plates, incubated for 18 h, and then treated with MHE and stimulated with LPS. After incubation, cells were harvested and resuspended in radioimmunoprecipitation assay (RIPA) lysis buffer (Millipore, Bedford, MA, USA) with a protease and phosphatase inhibitor cocktail (Roche, Basel, Switzerland) and centrifuged at 13,000 rpm for 5 min at 4 °C. The supernatant was collected and stored at −80°C. Cytosolic and nuclear fractions were isolated using NE-PER Nuclear and Cytoplasmic Extraction Reagents (Thermo Scientific, Rockford, IL, USA) following the manufacturer′s instructions.

### 4.8. Western Blot Analysis

Total protein of whole cell extracts or cytoplasmic and nuclear extracts was determined using Bradford reagent (Bio-Rad, Hercules, CA, USA). Equal amounts of protein were resolved on sodium dodecyl sulfate (SDS) gels by SDS-polyacrylamide gel electrophoresis (PAGE) and transferred to NC membranes. After blocking nonspecific sites with 3% BSA, membranes were incubated with each primary antibody (1:1000 dilution) and its corresponding secondary antibody (1:5000 dilution) and detected with SuperSignal West Femto Chemiluminescent Substrate (Thermo Scientific).

### 4.9. RNA Extraction and Real-Time RT-PCR

Total cellular RNA was extracted from RAW 264.7 cells using an easy-BLUE™ RNA Extraction Kit (iNtRON Biotechnology) following the manufacturer′s procedure. Total RNA (1 μg) was converted to cDNA using RevoScript™ RT PreMix (iNtRON Biotechnology, Sungnam, Korea). The oligonucleotide primers for real-time RT-PCR used with mouse macrophage cDNA are listed in Table 1. The reactions were conducted in triplicate with a total volume of 20 μL, comprised of 0.3 μM of each primer, 10 μL of AccuPower^®^ 2X Greenstar qPCR Master Mix (Bioneer, Daejeon, Korea), and 2 μL of template DNA. PCR was used to detect TNF-α, IL-6, IL-1β, iNOS, COX-2, HO-1, and β-actin following 40 cycles of 94 °C for 15 s and 60 °C for 1 min [52]. Amplification and analyses were performed using a QuantStudio 6 Flex Real-Time PCR System (Thermo Scientific). Samples were compared using the relative CT method. Individual transcripts in each sample were assayed three times and normalized to β-actin mRNA.

### 4.10. Chromatographic Conditions

The chromatographic analysis was conducted according a modified version of the process previously described [48]. MHE standardization was performed using HPLC fingerprinting with chemical standards of vitexin, ecdysterone (Sigma-Aldrich), and α-spinasterol (ChemFaces Biochemical, Wuhan, Hubei, China). Standard solutions were prepared by dissolving 1 mg/mL of each marker component in 100% methanol. MH powder was weighed accurately and dissolved in methanol at a concentration of 5 mg/mL for analysis. The HPLC-DAD system (Hitachi Co., Tokyo, Japan) consisted of a pump (L-2130), autosampler (L-2200), column oven (L-2350), and diode array UV-VIS detector (L-2455). Output signals from the detector were recorded with EZChrom Elite software (Hitachi Co., Tokyo, Japan). For the sample analysis, an OptimaPak C18 column (5 μm, 4.6 × 250 mm, RS Tech, Daejeon, Korea) was used, and the UV wavelength was set to 254 and 330 nm. The column temperature was maintained at 40 °C. The sample injection volume was 10 μL. The mobile phase consisted of water with 0.1% acetic acid (A) and acetonitrile with 0.1% acetic acid (B) at a flow rate of 1.0 mL/min with the following gradient flow: 10% B for 0–10 min, 10%–45% B for 10–55 min, 45%–50% B for 55–56 min, and 50%–100% B for 56–60 min. Peak identification was based on the UV spectra and retention times of each marker component from the MHE.

### 4.11. Statistical Analysis

All data are presented as the means ± standard deviation (SD) of at least three independent experiments. A one-way analysis of variance (ANOVA) followed by Dunnett’s test was used to determine significant differences between each treated group and the negative control (LPS group). *p* < 0.01 (*) and *p* < 0.001 (**) were considered to indicate statistical significance.

## 5. Conclusions

Our results demonstrate that MHE exhibits anti-inflammatory activity via inhibition of LPS-induced inflammatory mediators, including NO, inflammatory cytokines, and iNOS, in macrophages. These effects occur by blocking NF-κB activation via inhibition of IκBα degradation and down-regulation of p38 and JNK MAPK phosphorylation. In addition, the effect of MHE on HO-1 expression can be attributed to a reduction in inflammatory factors. Furthermore, MHE exerts the inhibitory effect on inflammatory cytokine production in mouse primary macrophages. These results imply that MHE could be developed as a new anti-inflammatory therapeutic herbal medicine after further *in vivo* studies.

## Figures and Tables

**Table 1:** Primers used for real-time RT-PCR.

Target Gene	Primer Sequence
TNF-α	F: 5′-TTCTGTCTACTGAACTTCGGGGTGATCGGTCC-3′
	R: 5′-GTATGAGATAGCAAATCGGCTGACGGTGTGGG-3′
IL-6	F: 5′-TCCAGTTGCCTTCTTGGGAC-3′
	R: 5′-GTGTAATTAAGCCTCCGACTTG-3′
IL-1β	F: 5′-ATGGCAACTGTTCCTGAACTCAACT-3′
	R: 5′-CAGGACAGGTATAGATTCTTTCCTTT-3′
iNOS	F: 5′-GGCAGCCTGTGAGACCTTTG-3′
	R: 5′-GCATTGGAAGTGAAGCGTTTC-3′
COX-2	F: 5′-TGAGTACCGCAAACGCTTCTC-3′
	R: 5′-TGGACGAGGTTTTTCCACCAG-3′
HO-1	F: 5′-TGAAGGAGGCCACCAAGGAGG-3′
	R: 5′-AGAGGTCACCCAGGTAGCGGG-3′
β-actin	F: 5′-AGAGGGAAATCGTGCGTGAC-3′
	R: 5′-CAATAGTGATGACCTGGCCGT-3′

F, forward; R, reverse.

## Abbreviations

The following abbreviations are used in this manuscript:

MH: Melandrii Herba
MHE: Melandrii Herba ethanol extract
LPS: lipopolysaccharide
NO: nitric oxide
ELISA: enzyme-linked immunosorbent assay
RT-PCR: reverse transcription polymerase chain reaction
NF: nuclear factor
MAPK: mitogen-activated protein kinases
HO: heme oxygenase
TNF: tumor necrosis factor
IL: interleukin
iNOS: inducible nitric oxide synthase
COX: cyclooxygenase
IκBα: inhibitor of NF-κB alpha
ERK: extracellular regulated kinase
JNK: c-Jun N-terminal kinase
PG: prostaglandin
ATCC: American Type Culture Collection
FBS: fetal bovine serum
BSA: bovine serum albumin
CCK: cell counting kit
NC: Nitrocellulose
PBS: phosphate-buffered saline
RIPA: radio immunoprecipitation assay
SDS: sodium dodecyl sulfate
PAGE: polyacrylamide gel electrophoresis
HPLC: high-performance liquid chromatography
SD: standard deviation
ANOVA: analysis of variance
